# Diversity in a Cold Hot-Spot: DNA-Barcoding Reveals Patterns of Evolution among Antarctic Demosponges (Class Demospongiae, Phylum Porifera)

**DOI:** 10.1371/journal.pone.0127573

**Published:** 2015-06-19

**Authors:** Sergio Vargas, Michelle Kelly, Kareen Schnabel, Sadie Mills, David Bowden, Gert Wörheide

**Affiliations:** 1 Department of Earth- & Environmental Sciences, Palaeontology and Geobiology, Ludwig-Maximilians-Universtität München, Richard-Wagner Str. 10, D-80333, München, Germany; 2 National Centre for Coasts and Oceans, National Institute of Water and Atmospheric Research, Private Bag 99940, Newmarket, Auckland, 1149, New Zealand; 3 National Centre for Coasts and Oceans, National Institute of Water and Atmospheric Research, Private Bag 14901, Wellington, New Zealand; 4 GeoBio-Center^LMU^, Richard-Wagner Str. 10, D-80333, München, Germany; 5 Bavarian State Collections of Palaeontology and Geology, Richard-Wagner Str. 10, D-80333, München, Germany; Victoria University Wellington, NEW ZEALAND

## Abstract

**Background:**

The approximately 350 demosponge species that have been described from Antarctica represent a faunistic component distinct from that of neighboring regions. Sponges provide structure to the Antarctic benthos and refuge to other invertebrates, and can be dominant in some communities. Despite the importance of sponges in the Antarctic subtidal environment, sponge DNA barcodes are scarce but can provide insight into the evolutionary relationships of this unique biogeographic province.

**Methodology/Principal Findings:**

We sequenced the standard barcoding COI region for a comprehensive selection of sponges collected during expeditions to the Ross Sea region in 2004 and 2008, and produced DNA-barcodes for 53 demosponge species covering about 60% of the species collected. The Antarctic sponge communities are phylogenetically diverse, matching the diversity of well-sampled sponge communities in the Lusitanic and Mediterranean marine provinces in the Temperate Northern Atlantic for which molecular data are readily available. Additionally, DNA-barcoding revealed levels of *in situ* molecular evolution comparable to those present among Caribbean sponges. DNA-barcoding using the Segregating Sites Algorithm correctly assigned approximately 54% of the barcoded species to the morphologically determined species.

**Conclusion/Significance:**

A barcode library for Antarctic sponges was assembled and used to advance the systematic and evolutionary research of Antarctic sponges. We provide insights on the evolutionary forces shaping Antarctica's diverse sponge communities, and a barcode library against which future sequence data from other regions or depth strata of Antarctica can be compared. The opportunity for rapid taxonomic identification of sponge collections for ecological research is now at the horizon.

## Introduction

Sponges are conspicuous and, in some cases, dominant members of the rich invertebrate communities that inhabit the Antarctic shelf [1–4]. Large sponges are key structural components of the Antarctic benthos, reaching high abundances and biomass [5], serving as structural frameworks for other filter feeders, and providing refuge for the juvenile and adult stages of numerous organisms [6–9]. In terms of species richness, Antarctic sponge assemblages are comparable to some tropical faunas (e.g. the western Indian Ocean, the Caribbean Sea [10]). The approximately 350 sponge species reported from the Antarctic shelf to date constitute a distinct faunistic assemblage characterized by its high species-level endemism and generic cosmopolitanism [3,11].

The high endemicity levels observed among Antarctic sponges have been hypothesized to be the result of the continent's and surrounding oceans' geological history and prolonged geographic isolation [3,11]. Antarctica separated from Gondwana approximately 140 million years ago and became progressively isolated from other land masses [12]. By the end of the Eocene, the opening of the Drake Passage broke Antarctica's last connection with South America and triggered the onset of the Antarctic Circumpolar Current (ACC) [13]. Cooling and the development of the ACC during the Oligocene [13] further isolated the Antarctic biota, likely promoting speciation in the marine realm [14,15]. *In situ* diversification in Antarctica has been documented for a number of marine invertebrates [16–20], and can be similarly assumed in sponges which typically produce short-living planktonic larvae [11].

Despite their ecological importance, Antarctic sponges have not been included in molecular phylogenetic or DNA-barcoding studies of the phylum Porifera, due in part to the difficulty of access for collection and the harshness of the environment. [21] found that only 2% of all sponge species reported for Antarctica had DNA sequence data, and to date only a handful of studies have dealt with the molecular systematics of selected Southern Ocean poriferans (e.g.[22]). The lack of Antarctic sponge sequences limits the evaluation of hypotheses regarding their macro-ecology and evolutionary history. DNA-barcoding [23] is proposed to rapidly sequence a representative set of Antarctic sponges. Here, we use a community-based approach (sensu [24]) to barcode the shelf and slope sponge associations occurring in the Ross Sea, Antarctica. We use the set of barcodes to determine the phylogenetic diversity of the Ross Sea sponge assemblage and compare it with the sponge phylogenetic diversity of well-sampled tropical and subtropical regions (e.g. the Mediterranean, the Lusitanic, the Tropical North-West Atlantic) for which sponges sequences are readily available in public data repositories (e.g. NCBI GenBank [25]). Our barcode dataset provides first insights into the evolutionary processes that led to Antarctica's high sponge diversity and endemism, and reveal areas of taxonomic uncertainty in need of further research.

## Results

### Phylogeny and phylogenetic diversity of Antarctic sponges

**Fig 1:**
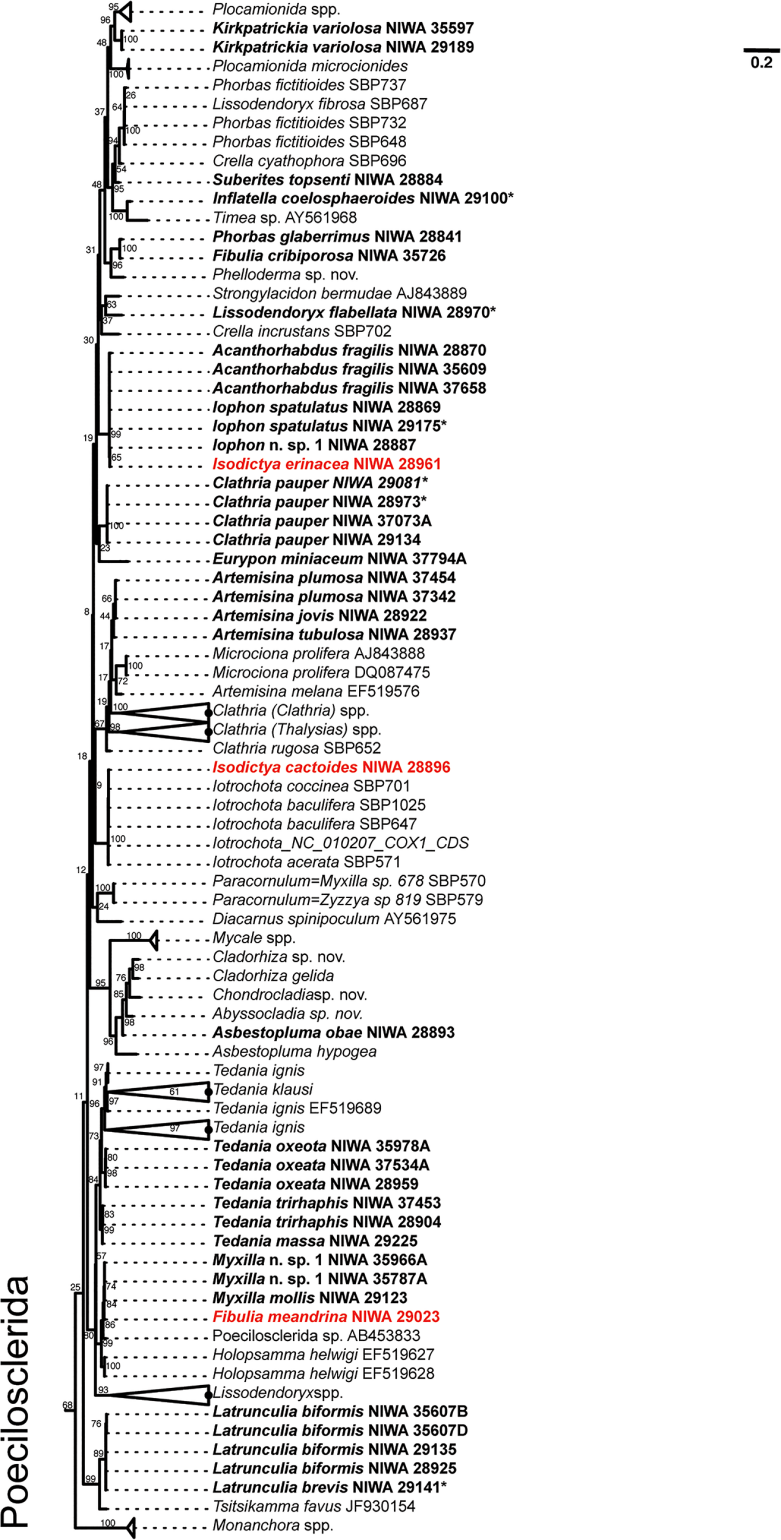
COI maximum likelihood phylogeny of Antarctic sponges (in bold face) belonging Order Hadromerida, Haplosclerida, Halichondrida, Spirophorida, Poecilosclerida (non-chelae bearing). For visualization, subtrees containing Antarctic sponges were pruned from the complete phylogenetic tree that included all sequences analyzed (i.e. GenBank + sequences from this study). Orders are indicated for each subtree. Bootstrap support is given near each branch of the tree. Specimens originally classified as a different species using morphology and reclassified after DNA-barcoding or presenting molecular-morphological discrepancies are in red (see Table 1). Specimens belonging the Spirophorida were sequenced for this study but were already published by Szitenberg et al. 2013.

**Fig 2:**
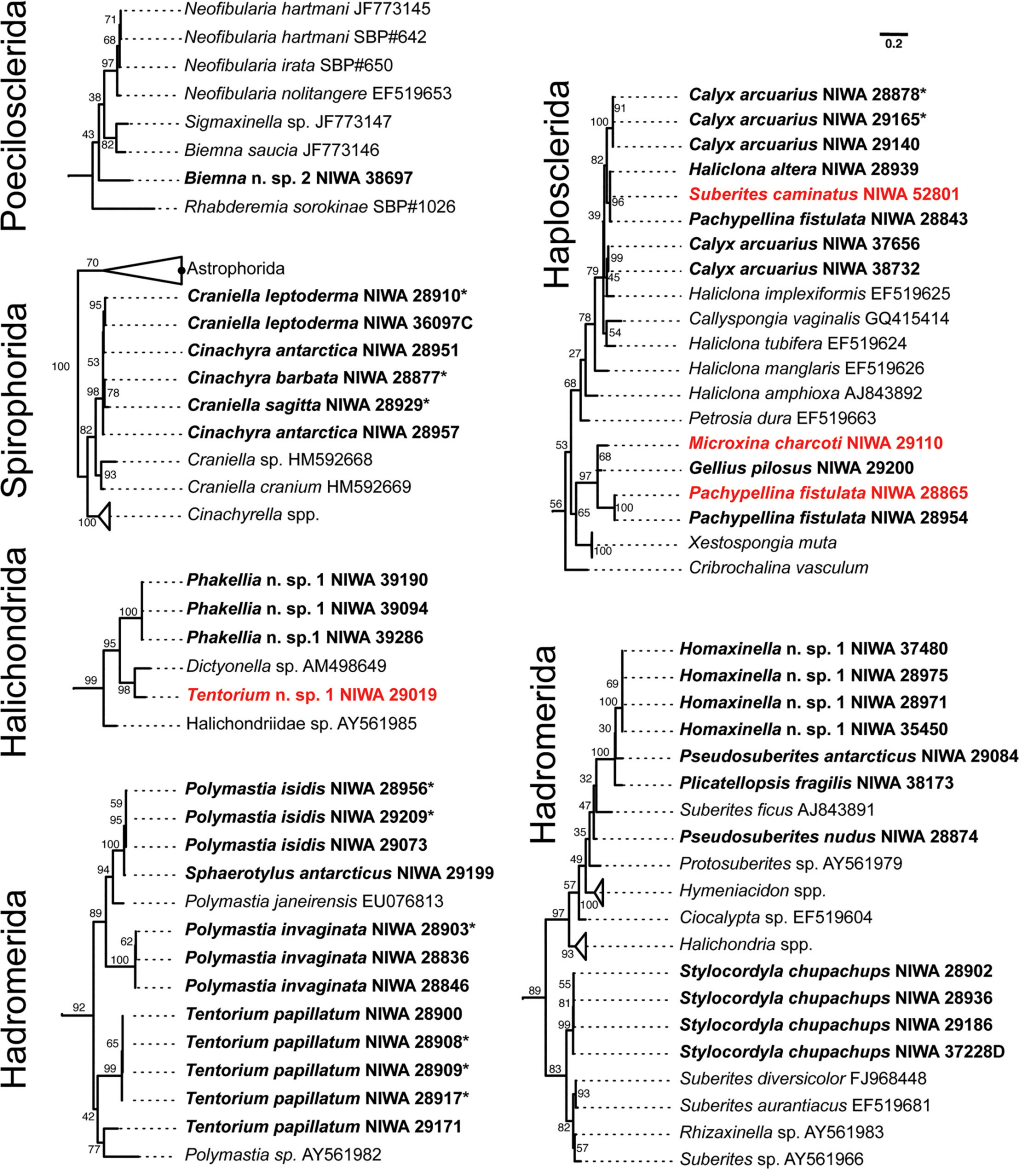
COI maximum likelihood phylogeny of Antarctic sponges (in bold face) belonging Order Poecilosclerida (chelae-bearing). For visualization, subtrees containing Antarctic sponges were pruned from the complete phylogenetic tree that included all sequences analyzed (i.e. GenBank + sequences from this study). Bootstrap support is given near each branch of the tree. Specimens originally classified as a different species using morphology and reclassified after DNA-barcoding or presenting molecular-morphological discrepancies are in red (see Table 1).

Sampled Antarctic sponges nest within several nominal demosponge orders, namely: Hadromerida, Halichondrida, Haplosclerida, Poecilosclerida and Spirophorida (Figs 1 and 2). Hadromerida contains two major clades, the first containing *Sphaerotylus antarcticus* Kirkpatrick, *Polymastia isidis* Thiele, and *P*. *invaginata* Kirkpatrick (Polymastiidae), and *Tentorium papillatum* (Kirkpatrick) (Suberitidae) and *Polymastia* sequences harvested from GenBank, and the second containing predominantly Suberitidae genera *Homaxinella*, *Pseudosuberites*, *Plicatellopsis*, together with *Stylocordyla* (Stylocordylidae), and with halichondrid sequences harvested from GenBank. *Tentorium papillatum* was not monophyletic due to the inclusion of a GenBank sequence attributed to the genus *Polymastia*.

The Antarctic samples from the order Halichondrida was represented by 4 new sequences, three of which are described operatively as *Phakellia* sp. 1 (thin veined bristly fan) (Halichondrida, Axinellidae), also known from the Chatham Rise and Christable Seamount to the south of New Zealand. The species forms a distinctive tall thin oval fan, with sinuous veins on the surface which is ribbed and bristly. The spicules are long styles and long highly contorted vermicular oxeas. This species resembles the foliaceous specimens of *Phakellia vermiculata* (Bowerbank) sensu Koltun (1964), but differs in having much longer vermicular oxeas. It also superficially resembles *Axinella antarctica* (Koltun), first described as *Phakellia antarctica*, with long styles and long highly contort, vermicular oxeas, but the morphology of the latter is globular. These three specimens formed a sister clade to a species of *Dictyonella* (Halichondrida, Dictyonellidae).

Poecilosclerida also form two major clades, species of the families Desmacellidae (*Biemna* sp. 2) and Rhabderemidae (Fig 1) were not closely related to chelae-bearing poecilosclerids (Fig 2). Poecilosclerid *Kirkpatrickia variolosa* Kirkpatrick is nested within the Hymedesmiidae as represented by species of *Plocamionida* and *Phorbas* spp. (Fig 1). It is interesting to note that NIWA 28884 was identified as Burton’s *Suberites topsenti* (Burton), but the specimen contains mycalostyles and has areolate porefields, also noted in the description of the original specimens, so is definitely not a member of the hadromerid family Suberitidae. This is confirmed by the hypothesised affinity of this specimen with *Phorbas* spp (Hymedesmiidae) which can also have areolate porefields.

*Phorbas glaberrimus* (Topsent) was not related to other species of *Phorbas* available in GenBank but to *Fibulia cribriporosa* (Burton) from the Ross Sea, and *Lissodendoryx flabellata* Burton was not related to other species of the genus included in the phylogenetic analysis. Species of *Iophon* (Acarnidae) formed a highly supported clade with acarnid *Acanthorhabdus fragilis* Burton and *Isodictya erinacea* (Topsent) (Isodictyidae) this last species was not related to *Isodictya cactoides* (Kirkpatrick) which was included in a clade with species of *Iotrochota* (Iotrochotidae). All specimens of *Clathria pauper* (Brøndsted) formed a highly supported monophylum that was not related to other members of the genus *Clathria*. Within this genus, the Antarctic species of *Artemisina* formed a clade and were not related to *Artemisina melana* van Soest from GenBank. The genus *Tedania* was monophyletic, with *Tedania oxeata* Topsent more closely related to *Tedania ignis* (Duchassaing & Michelotti) *+ Tedania klausi* Wulff than to other Antarctic species (i.e. *Tedania trirhaphis* Koltun and *Tedania massa* Ridley & Dendy). Antarctic *Myxilla* formed a highly supported clade. *Latrunculia* spp. and *Tsitsikamma favus* Samaai & Kelly were monophyletic and highly supported.

Finally, Spirophorida formed a monophylum with a highly supported clade of mixed *Craniella* and *Cinachyra* species (see also [26]). *Craniella* cf. *leptoderma* (Sollas) forms a sister clade to *Cinachyra antarctica* (Carter), *Cinachyra barbata* Sollas and *Craniella sagitta* (Lendenfeld).

**Fig 3:**
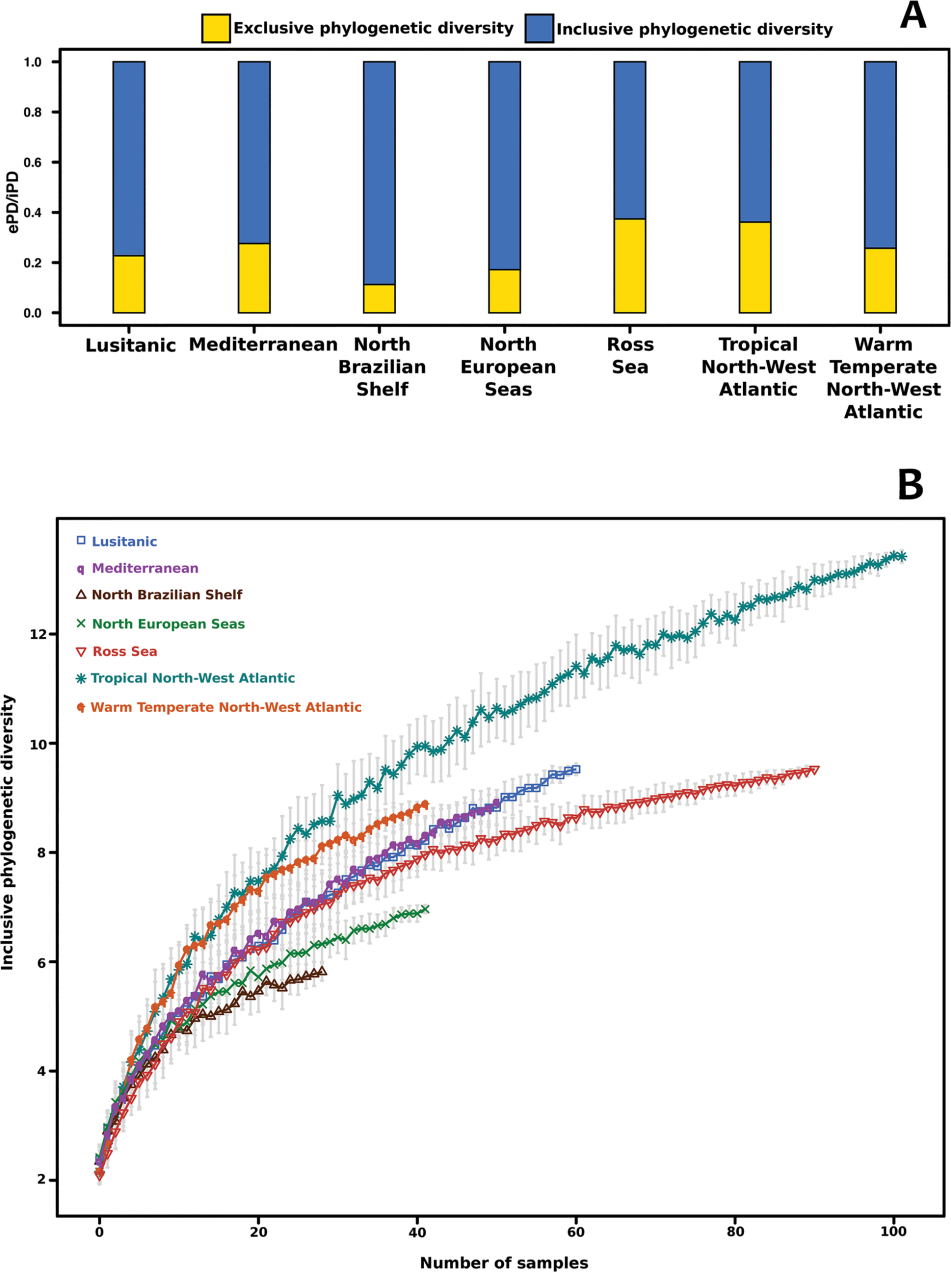
Sponge phylogenetic diversity for seven marine provinces in the Atlantic, the Mediterranean, and the Ross Sea. Upper figure: exclusive and inclusive phylogenetic diversity for each province. Lower figure: rarified inclusive phylogenetic diversity per marine province analyzed.

The rarified inclusive phylogenetic diversity (PD_I_) curves for seven extensively sampled Marine Provinces are shown in Fig 3. The North Brazilian Shelf was the least diverse marine province sampled while the Tropical North West Atlantic showed the highest PD_I_ values. The Ross Sea had intermediate PD_I_ values comparable to the Lusitanic and Mediterranean Sea provinces.

Interestingly, the amount of phylogenetic diversity attributable to *in situ* evolution (Fig 3) within an area was maximal for the Ross Sea and the Tropical North West Atlantic, where exclusive Phylogenetic Diversity (PD_E_) accounted for approximately 35% of the PD_I_ of both areas. PD_E_ was minimal for North Brazilian Shelf and North European Seas (11% and 17% respectively) and reached intermediate values (17–27%) for the Lusitanic, Mediterranean Sea and Warm-Temperate North-West Atlantic marine provinces.

### DNA-barcoding assignment power

**Fig 4:**
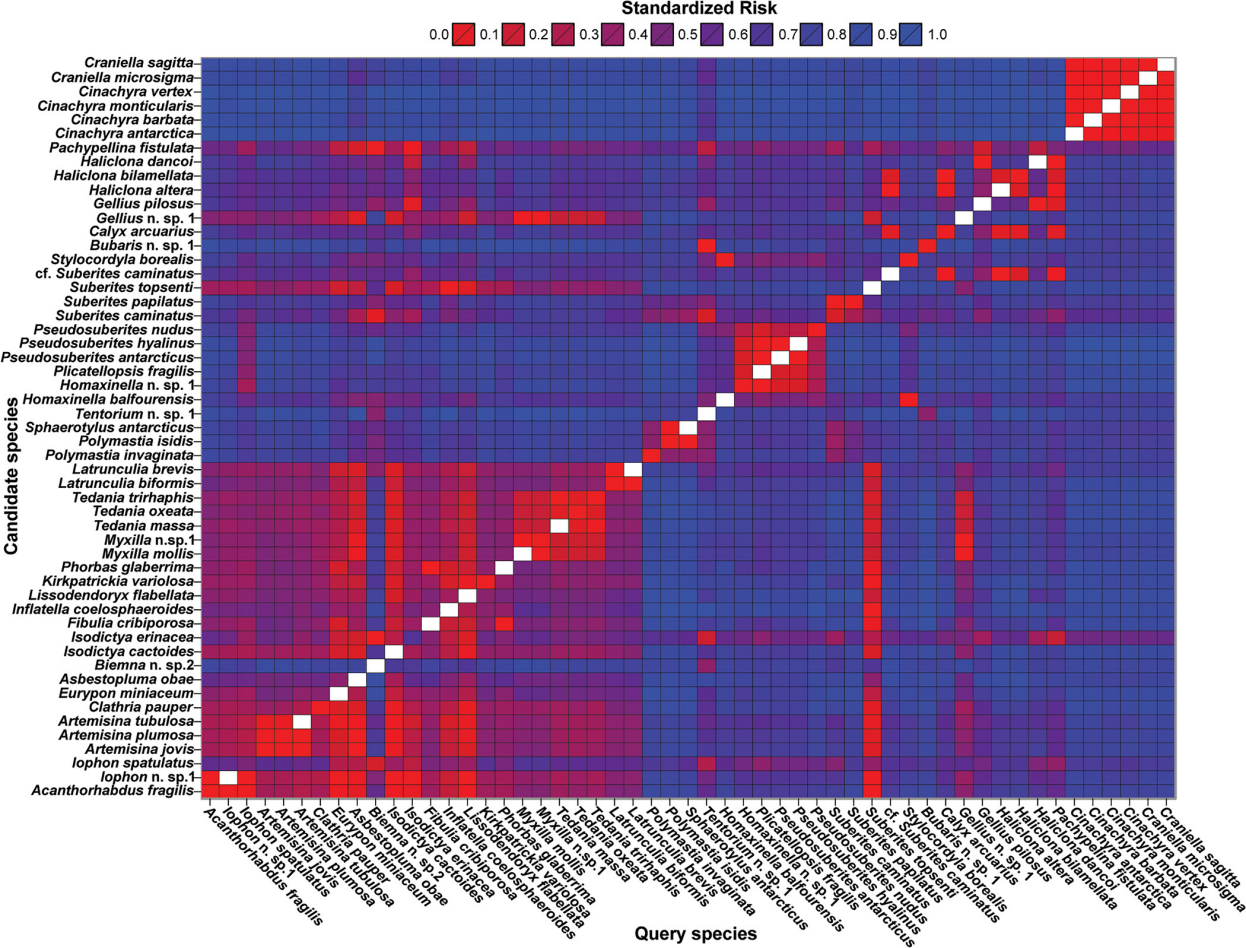
Classification accuracy (assignment risk) of the standard DNA-barcoding fragment (COI) for 51 species of Antarctic sponges. Species were taxonomically identified using morphological characters and the assignment risk was assessed using leave-one-out validation. The risk values from different runs were range standardized to make them comparable. In general, a query species is assigned to the candidate species of minimum risk. Candidate species refer to sequences in the DNA-barcoding database used to classify undetermined (query) sequences. Query species are the true species of the queried sequence used to test the classification accuracy of the COI for sponge determination. The Heatplot was done in R using the results obtained from SSA.

The Segregating Sites Algorithm (SSA) correctly assigned approximately 54% of the species in the Ross Sea region samples. Among the remaining species, about 47% were found to be either potential misidentifications or groups of specimens which are known to require taxonomic revision (see Discussion), and 53% can be attributed to the incompleteness of the candidate species database. Fig 4 shows the standardized assignment risk resulting from the leave-one-out cross validation SSA trials. In general, the assignment risk using the standard COI barcoding fragment remained low to moderate within families or orders and increased between them. In the case of specimens for which only one sequence was available, SSA was able to assign the queries to related genera in the same family (e.g. *Cinachyra* as *Craniella*, *Iophon* as *Acanthorhabdus*, and *Plicatellopsis* as *Homaxinella*) or clade (e.g. *Fibulia* as *Phorbas* and vice versa).

## Discussion

We have presented the results of the first DNA-barcoding campaign directed towards sequencing sponges collected in the Ross Sea region, Antarctica. In terms of the coverage reached by our barcoding campaign, approximately 60% of the demosponge species collected during NIWA's 2004 BioRoss and 2008 IPY-CAML expeditions were successfully barcoded. In this respect, DNA-barcoding was successful in rapidly gathering information about Antarctic sponge communities that can be used for phylogenetic inference (Figs 1 and 2) and phylo-diversity comparisons (Fig 3), or for sorting large collections and complement classical taxonomic work.

The inferred COI gene-tree (Figs 1 and 2) clearly pointed to specimens in need of deeper taxonomic examination (Table 1). Within Haplosclerida, this phylogeny revealed a rather diverse, highly supported clade joining together specimens of *Gellius pilosus* Kirkpatrick, *Pachypellina fistulata* (Kirkpatrick) and two specimen initially identified as *Haliclona dancoi* (Topsent) and *Isodictya erinacea* (Topsent) (Poecilosclerida). Regarding these specimens, *I*. *erinacea* had no chelae but microoxeas diagnostic of *Microxina*, a member of order Haplosclerida [27], and *H*. *dancoi* was in fact a specimen of *P*. *fistulata*. It is interesting to note that Burton (1929) (cited in [28]) considered *P*. *fistulata* a juvenile of *H*. *dancoi* because of their morphological similarity, thus it is likely that these species are confused on a regular basis. In contrast to the examples above, all the barcoded specimens of *Calyx arcuarius* (Topsent) had toxas and oxeas in accordance with the species description [28]. The oxeas of these specimens were somewhat different, being either stout or more delicate, pointing to the need for further taxonomic work on these specimens, which could represent an assemblage of cryptic species. Two more cases worth noting, are the exclusion of *C*. *pauper* from *Clathria* already suggested by de Laubenfels [29] and the inclusion of *Suberites (Laxosuberella) topsenti* in Poecilosclerida, foreseen by MK who annotated this species as “related to *Semisuberites*” because its “tylostyles are mycalostyles”; both hypotheses are here corroborated and prompt a future taxonomic reclassification of these taxa. Within Poecilosclerida, the genus *Isodictya* was not monophyletic with *I*. *erinacea* allied with *Iophon* spp. and *Acanthorhabdus fragilis* and *I*. *cactoides* included in a clade with species of *Iotrochota*. The DNA barcodes of these species should be confirmed to further evaluate the monophyletic/non-monophyletic status of *Isodictya*. Table 1 summarizes potential misidentifications, contaminations or cases in which DNA-barcoding prompted a reevaluation of morphology-based taxonomic determination.

**Table 1:** Examples of potential misidentifications, contaminations or cases in which a taxonomic re-evaluation resulted in a corroboration of the molecular results obtained using DNA barcoding.

Original taxonomic identification	Molecular results	Morphological observations	Revised taxonomic identification
*Gellius* n. sp. 1 (NIWA 29023)	Included in a clade with *Myxilla* specimens	After re-examination of the specimen it was concluded that the sample is in Myxillina, and is most closely comparable to *Fibulia maeandrina* (Kirkpatrick, 1907), but that the taxonomic determination was difficult due to the macerated state of the specimen and a resulting lack of chelae.	*Fibulia maeandrina*
*Haliclona dancoi* (NIWA 28865)	Included in a clade with *Pachypellina fistulata*	Originally identified as *H*. *dancoi* due to smaller size of oxeas than other *P*. *fistulata* specimens. Reclassified as *P*. *fistulata* after re-examination of the specimen and comparison to specimens of *H*. *dancoi*	*Pachypellina fistulata*
*Isodyctia erinacea* (NIWA 29110)	Included in a clade with *Pachypellina fistulata* and *Gellius pilosus*	Reclassified as *Microxina charcoti* after re-examination of the specimen.	*Microxina charcoti*
cf. *Suberites caminatus* (NIWA 52801)	Included in a clade with *Haliclona altera* and *Pachypellina fistulata*, nested within other Haplosclerida	Specimen found in same lot as hexactinellid *Bathydorus spinosus*. Re-examination of the subsampled specimen confirms the identification as *Suberites caminatus*. See footnote 1. **Barcode to be confirmed.**	*Suberites caminatus*
Undet sp. 1 (NIWA 29019)	Included in a clade with *Dictyonella* sp., and sister group to *Phakellia* n. sp. 1	Originally identified as *Tentorium* n. sp. 1 (MK: huge strongyloxeas, towering, conical, Antarctica). Re-examination of the subsampled specimen confirms the identification. See footnote 2. **Barcode to be confirmed.**	*Tentorium* n. sp. 1
*Isodictya erinacea* (NIWA 28961)	Included in clade with *Acanthorabdus fragilis* and *Iophon* spp. (Family Acarnidae).	Re-examination of the subsampled specimen confirms the identification as *Isodictya erinacea*. **Barcode to be confirmed.**	*Isodictya erinacea*
*Isodictya cactoides* NIWA 28896	Included in clade with *Iotrochota* spp. (Family Iotrochotidae)	Re-examination of the subsampled specimen confirms the identification as *Isodictya cactoides*. **Barcode to be confirmed.**	*Isodictya cactoides*

**Footnote 1.** The original sample NIWA 37922 is a specimen of *Bathydorus spinosus* Schulze, a hexactinellid species. A second species was found in the original specimen lot, removed, and given the new accession number NIWA 52801. Re-examination of NIWA 52801 confirms the identity as *Suberites caminatus* and not as a haplosclerid taxon. All artifact material in the containers have been checked for contaminating taxa. The original subsampled specimen lot is clear of all additional contaminants.

**Footnote 2.** NIWA 29019 was identified as a species of *Tentorium* (Hadromerida, Polymastiidae) as it is very similar to other species such as *T*. *semisuberites* (Schmidt) forming a vertical cylinder-like palisade of strongyloxeas. NIWA 29019 differs from *T*. *semisuberites* in that it forms a single stiff spike arising from the substrate. The spicules are extremely long subtylostyles and strongyloxeas up to 4000 μm, with some shorter subtylostyles. Dictyonellidae contains some genera with strongyloxea-like spicules but in a very different arrangement to that of NIWA 29019. While this barcode discrepancy may be taken to suggest an alternative classification for this taxon, we find the similarity to species of *Tentorium* overwhelming, and the suggestion that the taxon is a dictyonellid taxa, extremely unlikely.

Sponge taxonomy and nomenclature are complicated and can easily obfuscate the study of diversity patterns among sponges [30]. It has been suggested that the diversity of Antarctica's sponge communities is comparable to that of some tropical ecosystems, like the Caribbean [10]. The analysis of the phylogenetic diversity of the Ross Sea collection revealed PD_I_ values comparable to those observed in subtropical marine provinces (i.e. Lusitanic and Mediterranean marine provinces) and higher than those of some marine provinces in tropical realms (e.g. North Brazilian Shelf). This result corroborates previous observations pointing towards a high diversity of sponges in Antarctic waters. Given that the Ross Sea represents only one of the six Marine Ecoregions of the World (MEOW [31]) that are grouped within the Continental High Antarctic marine province, whereas the Lusitanic and Mediterranean Sea MPs include three and seven well-sampled MEOWs, respectively, it is very likely that the inclusion of a more comprehensive sample of sponges from other areas in the Continental High Antarctic marine province will result in Antarctic PD_I_ values comparable to those of tropical marine provinces (e.g. Tropical North-West Atlantic); especially considering that circumpolarity in many Antarctic taxa has been rejected and is unlikely in sponges [3].

The high PD_E_ observed for the Ross Sea sample implies that a high proportion of substitution changes in the standard COI barcoding fragment can be attributed to *in situ* (within the Southern Ocean) evolutionary change [32]. This pattern of evolution can be the result of Antarctica's age and prolonged isolation [11,13], especially considering that the rates of evolution of Antarctic organisms are expected to be generally low [33,34]. It also favors, albeit indirectly, the hypothesis of an ancient (Gondwanan) origin followed by *in situ* diversification of Antarctic sponges. In agreement with this hypothesis, the inferred COI gene-tree included clades exclusively composed of Antarctic species (Fig 1) in Haplosclerida (e.g. *Calyx* + *Haliclona* + *Pachypellina* or *Microxina* + *Gellius* + *Pachypellina*), Hadromerida (e.g. *Plicatellopsis* + *Pseudosuberites* + *Homaxinella* or *Polymastia + Sphaerotylus*) and Spirophorida (*Cinachyra* + *Craniella*). However, other sponge groups (e.g. *Tedania* in Poecilosclerida or *Polymastia* in Hadromerida) revealed that more complex historical processes likely played a role in shaping demosponge diversity patterns in the Ross Sea, and presumably in Antarctica. The lack of a time-calibrated phylogeny and the unbalanced taxonomic and geographic sampling currently available complicates testing these hypotheses. Future studies, ideally based on a more extensive sample of specimens and covering various localities around the Antarctic continent and neighboring regions, will help to clarify the relative contribution that dispersal and vicariance processes have had in shaping Antarctic's large sponge diversity.

DNA-barcoding can be used to sort and classify new collections from a particular geographic region provided that there is a reliable candidate species database available for this task. Here, using the standard barcoding (COI) fragment and the SSA we were able to correctly identify about 54% of the sampled species. This accuracy is most likely over-conservative because specimens were not correctly classified due to their absence in the candidate species database, or because the specimen was originally assigned to a species but reassigned in a different taxon after DNA-barcoding. We considered these cases to be misidentifications even if DNA-barcoding correctly assigned the specimens to their correct taxon. In this study we also observed instances in which DNA-barcoding cannot correctly assign a specimen to its species because multiple species share identical or nearly identical barcodes (e.g. the species of *Latrunculia*, *Artemisina* and *Acanthorhabdus* and *Iophon*; Fig 2). In these cases, a probabilistic taxonomic assignment using DNA-barcodes like the one used here should result in a large uncertainty associated to the taxonomic ID for the specimens involved prompting the examination of the material using other taxonomic tools. Yet, despite the assignment power limitations of barcoding in Porifera, this technique represents an attractive methodology to speed-up taxonomic work in sponges. Considering that DNA-barcoding can be easily done for many taxonomic groups simultaneously by technical staff trained in general molecular biology techniques [35], this method has the potential for reducing a taxonomist's work load allowing specialists to focus only on specimens not accurately classified through barcoding. These specimens can either be species absent from the original candidate species database, misidentifications, groups with a complex taxonomy in need of detailed revisionary work or groups of species sharing identical barcode sequences (e.g. species of *Latruculia*; Fig 2). Additionally, DNA-barcoding can be used in conjunction with traditional morphological studies to simultaneously provide access to genotypic and phenotypic data for a (potentially) large number of specimens, promoting integrative taxonomic research and cross validation between both identification approaches, and provide further inroads into our understanding of the evolution of this challenging phylum.

## Material and Methods

### Sample collection

**Fig 5:**
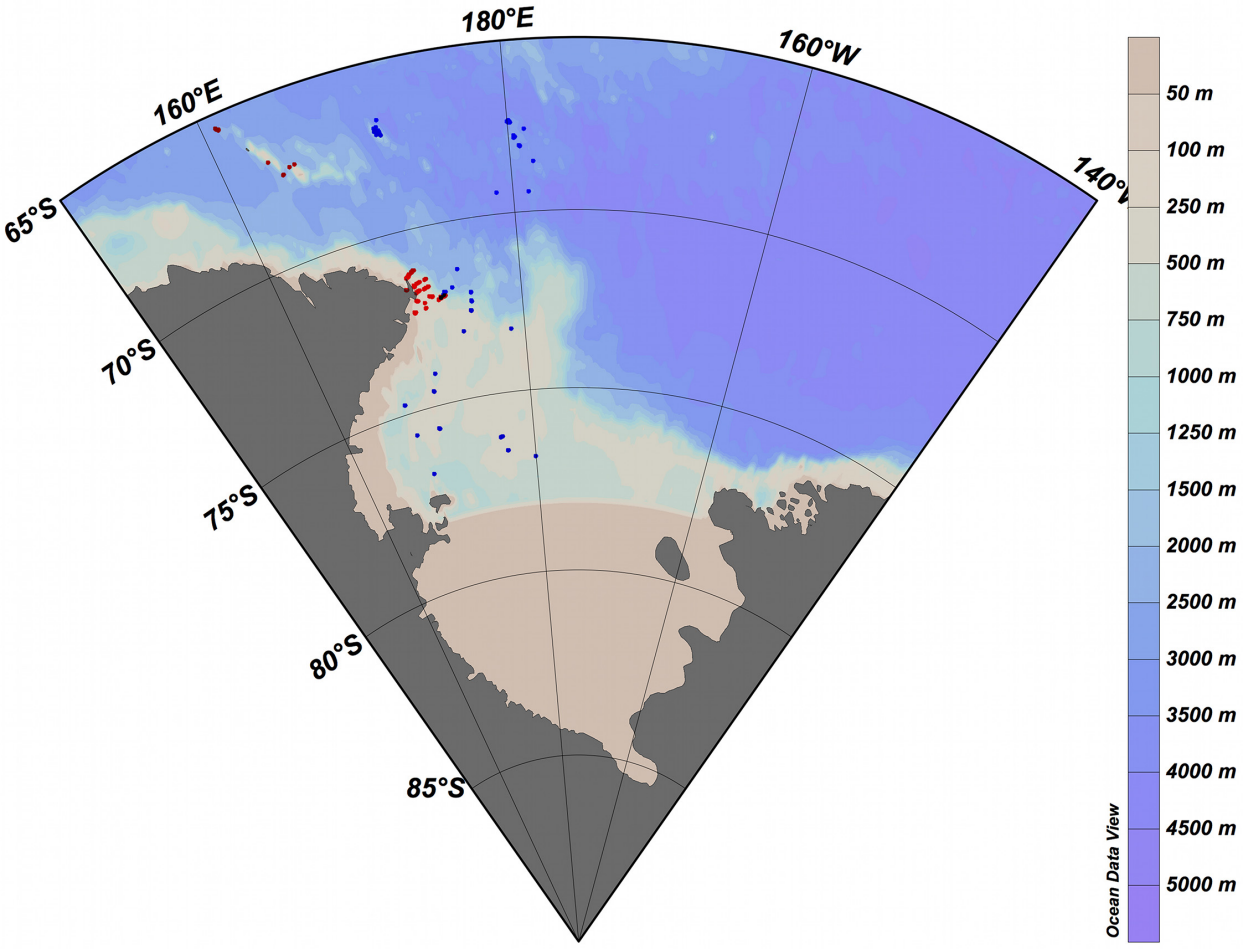
Distribution of the stations sampled during New Zealand's BioRoss (2004, TAN0402; red dots) and IPY-CAML (2008, TAN0802; blue dots) expeditions to the Ross Sea.

Sponges were collected by epibenthic sledge and trawls during New Zealand's BioRoss (2004, TAN0402, Antarctic Marine Living Resources Act 1981, Permit number: AMLR 04/001/NIWA/ZMFR [36]) and IPY-CAML (International Polar Year, Census of Antarctic Marine Life, 2008, TAN0802, Antarctic Marine Living Resources Act 1981, Permit number: AMLR 07/005/Tangaroa/ZMFR [37]) expeditions to the Ross Sea region of Antarctica (Fig 5; S1 Table). Sponges were sorted and frozen on board, and later transferred to, and stored in, 70% ethanol. Sponges were taxonomically determined by Michelle Kelly and accessioned into the National Institute of Water and Atmospheric Research (NIWA) Invertebrate Collection (NIC), Wellington, New Zealand. The study did not involve endangered or protected species. GPS data for all stations sampled during the TAN0402 and TAN0802 are provided in the S1 Table. For this study only demosponges were barcoded, the list of barcoded specimens with its associated collection data is provided as S2 Table.

### Molecular methods

Genomic DNA was extracted from pieces of pre-frozen or 99% ethanol-preserved sponge tissue using a modified [38] extraction protocol (see [35]). The standard barcoding fragment was initially amplified using primers dgLCO1490 and dgHCO2198 [39], however, using this primer set we mainly obtained sequences from non-target organisms, especially bacteria. In order to reduce the amplification of non-target organisms a new forward primer (SpongeCOI-F1: 5'-**ACATTTTGCTGCCGGTC**AGATAGGDACWGCNTTTA-3'), located ca. 33bp downstream from the dgLCO1490 and supplemented with an M13 tail (in bold face), was designed to specifically exclude bacteria. A quantitative evaluation of the new primer performance in terms of amplification success across different demosponge groups was not possible due to the low quality of the DNA extracted from the samples, which precluded attributing amplification failure to primer incompatibilities. However, using this new primer we exclusively amplified/sequenced sponge DNA (SV pers. obs.). We used a standard three-step PCR consisting of an initial denaturation step of 94°C for 3 minutes, 35–40 cycles consisting each of a denaturation step of 94°C for 30 seconds, an annealing step of 40°C for 30 seconds and an extension step of 72°C for 1 minute, and, finally, a last extension step of 72°C for 5 minutes. For samples that proved difficult to amplify we used a semi-nested approach in which a first, short PCR reaction (15–20 cycles; PCR program as above) using the primers dgLCO1490 and dgHCO2198 was re-amplified using primers SpongeCOI-F1+dgHCO2198 (35–40 cycles; PCR program as above). Negative controls were used for both DNA extractions and PCR reactions to monitor potential contaminations. If the semi-nested PCR approach was used, the negative control from the first PCR was also re-amplified. PCR products were excised from a 1.5% agarose gel (TAE) and purified using a modified freeze-squeeze method. Sequencing was carried out in both directions using primer M13-20F (5'-ACATTTTGCTGCCGGTC-3') and dgHCO2198 and the Big Dye Terminator version 3.1 (Applied Biosystems) chemistry. Sequencing reactions were cleaned-up using a standard ethanol-ammonium acetate precipitation following the BigDye Terminator 3.1 manual protocol and analyzed on an ABI 3730 Genetic Analyzer at the Sequencing Service of the Department of Biology, LMU München. Trace files were assembled in CodonCode Aligner (CodonCode Corporation) and the sponge origin of the sequences was confirmed using BLAST function on GenBank (www.ncbi.nlm.nih.gov). Sequences are deposited at EMBL under accession numbers LN850166 to LN850254 and the Sponge Barcoding Project (http://www.spongebarcoding.org/) accessions SBD 1166–1252.

### Phylogenetic methods and phylogenetic diversity

Newly obtained COI sequences from this study and demosponge COI sequences available from GenBank (Query: Porifera AND COI; Query date 16 Sept. 2011) were aligned in Seaview [40] using the Muscle algorithm (v3.8.31; [41]) with default settings. The resulting alignment was used to infer a bootstrapped Maximum Likelihood (ML) tree (1000 fast pseudo-replicates [42]) using RAxML 7.2.8 [43]. We used the GTR model of sequence evolution and accounted for among-site rate variation using a discrete gamma distribution with 4 categories. Bayesian phylogenetic analyses (available on request) were done in MrBayes 3.2.1 [44] under the same model settings as above but were not used further because the estimation of branch-lengths in a Bayesian context is problematic [45], and most of our analyses (see below) use the branch-lengths of the COI gene-tree.

The ML topology excluding duplicated sequences (i.e. RAxML “reduced” dataset) was used to estimate inclusive and exclusive molecular phylogenetic diversity (denoted PD_I_ and PD_E_, respectively [32]) for the Ross Sea shelf sponge samples and for several Marine Provinces (MPs sensu [31]) that have been extensively sampled for molecular studies (e.g. [46]). The inclusive molecular phylodiversity of a group corresponds to the length of the path connecting all members of the group, in this case all sponges assigned to an MP. Exclusive molecular phylodiversity is defined as the sum of the lengths of branches exclusively leading to members of the group of interest (see [32] for an explanatory figure). The inclusive molecular phylodiversity gives the amount of change accumulating from the root to the leaves of the tree while the exclusive molecular phylodiversity only provides information about the changes occurring along branches exclusively leading to members of a predefined group (see [32] for more information).

In order to calculate PD_I_ and PD_E_, each species in the ML tree was annotated as present/absent in the Northern European Seas, the Lusitanian, the Mediterranean Sea, and the Warm Temperate Northwest Atlantic marine provinces in the Temperate Northern Atlantic Marine Realm, and the Tropical Northwest Atlantic and North Brazilian Shelf marine provinces in the Tropical Atlantic Marine Realm. Species lists for these marine provinces were obtained from the World Porifera Database (WPD; www.marinespecies.org/porifera) and are provided as S1 Text because the lists available at the WPD are expected to change overtime as the database is refined and corrected. Species in the tree that did not belong to these provinces or that could not be annotated because the sequenced specimen is not classified to species-level (i.e. species such as, for instance, *Clathria* sp.) were left unannotated and were trimmed for the calculus of the PD_I_ and PD_E_ (see below).

The PD_I_ of a marine province was obtained by trimming all branches leading to species not occurring in the marine province of interest and computing the length of the resulting tree, which contains only those species in the marine province of interest. For PD_E_ calculation, first all branches leading to species occurring in the marine province of interest were trimmed and the length of the resulting tree was computed. The PD_E_ can then be easily calculated by subtracting this tree length from the total tree length of the unmodified tree.

Direct PD_I_ comparisons between marine provinces are difficult due to the different sampling efforts historically allocated to them. We used rarefaction [47] to make comparisons between marine provinces with different sampling efforts. In brief, if *N* leaves on the ML tree are known to occur in a given marine provinces, we subsampled *k = {1*,…, *N}* leaves and estimated the PD_I_ of the taxon set *k*. Because there are *N choose k* ways to sample *k* taxa from a set of *N* taxa, we used Monte Carlo sampling to approximate the phylogenetic diversity for any given *k<N* [48]. For a fixed number of pseudoreplicates, *k* terminals were randomly chosen and the complement set *k'* was pruned from the ML tree. The inclusive phylogenetic diversity of the sample *k* (PD_I,k_) was determined as the difference between the total length of the ML tree and the length of the *k'*-pruned tree. The rarified phylogenetic diversity values were used for inter-marine province comparisons. In addition, for all marine provinces we estimated the PD_E_/PD_I_ ratio for the total sample of taxa available for a given marine province (i.e. *N*). A python script implementing the method outlined above is available on request from the first author.

### DNA-barcoding

In order to assess the assignment power of the COI barcode in the case of Antarctic sponge species, we used leave-one-out cross-validation and the Segregating Sites Algorithm (SSA [49] available at http://info.mcmaster.ca/TheAssigner/). The SSA uses a Bayesian approach to assign sequences of unknown taxonomic affiliation to a set of sequences of known taxonomic origin. Each COI sequence obtained from Ross Sea sponges was used as a query against a dataset consisting of the remaining sequences, and the assignment risk of the query to each species in the candidate species dataset was determined. The minimum-risk assignment is the posterior probability of assigning a query sequence S to species i weighted by a loss function which represents the loss associated with assigning S to species i when in fact S belongs to species k. The loss function in the case of the SSA algorithm is defined by the distance between the query sequence S and the consensus sequence of species i, k, etc. In this study, a species assignment was considered correct if the minimum risk candidate species matched the queried species or, in the case of species for which only one sequence was available, if the minimum risk candidate species matched the genus of the query species. To facilitate comparisons, after each leave-one-out trial the assignment risk (*R*) of the query sequence, *i*, to each sequence, *j*, in the dataset (*R*_*i*,*j*_) was range standardized using the formula *R* ^*s*^_*i*,*j*_ = (*R*_*i*,*j*_ *−min(R))/(max(R) + min(R))*.

## Supporting Information

S1 Table Geographic data of stations sampled during the TAN0402 and TAN0802 cruises. (CSV)

S2 Table Collection data for barcoded specimens. (XLS)

S1 Text Species assignment to the Marine Provinces of the World according to the World Porifera Database. (TXT)
