# Correction: Diversity in a Cold Hot-Spot: DNA-Barcoding Reveals Patterns of Evolution among Antarctic Demosponges (Class Demospongiae, Phylum Porifera)

**DOI:** 10.1371/journal.pone.0133096

**Published:** 2015-07-14

**Authors:** Sergio Vargas, Michelle Kelly, Kareen Schnabel, Sadie Mills, David Bowden, Gert Wörheide

Fig 1 and Fig 2 are inadvertently switched. Fig 1 should be Fig 2 and Fig 2 should be Fig 1. The authors have provided the correct figures here.

**Fig 1:**
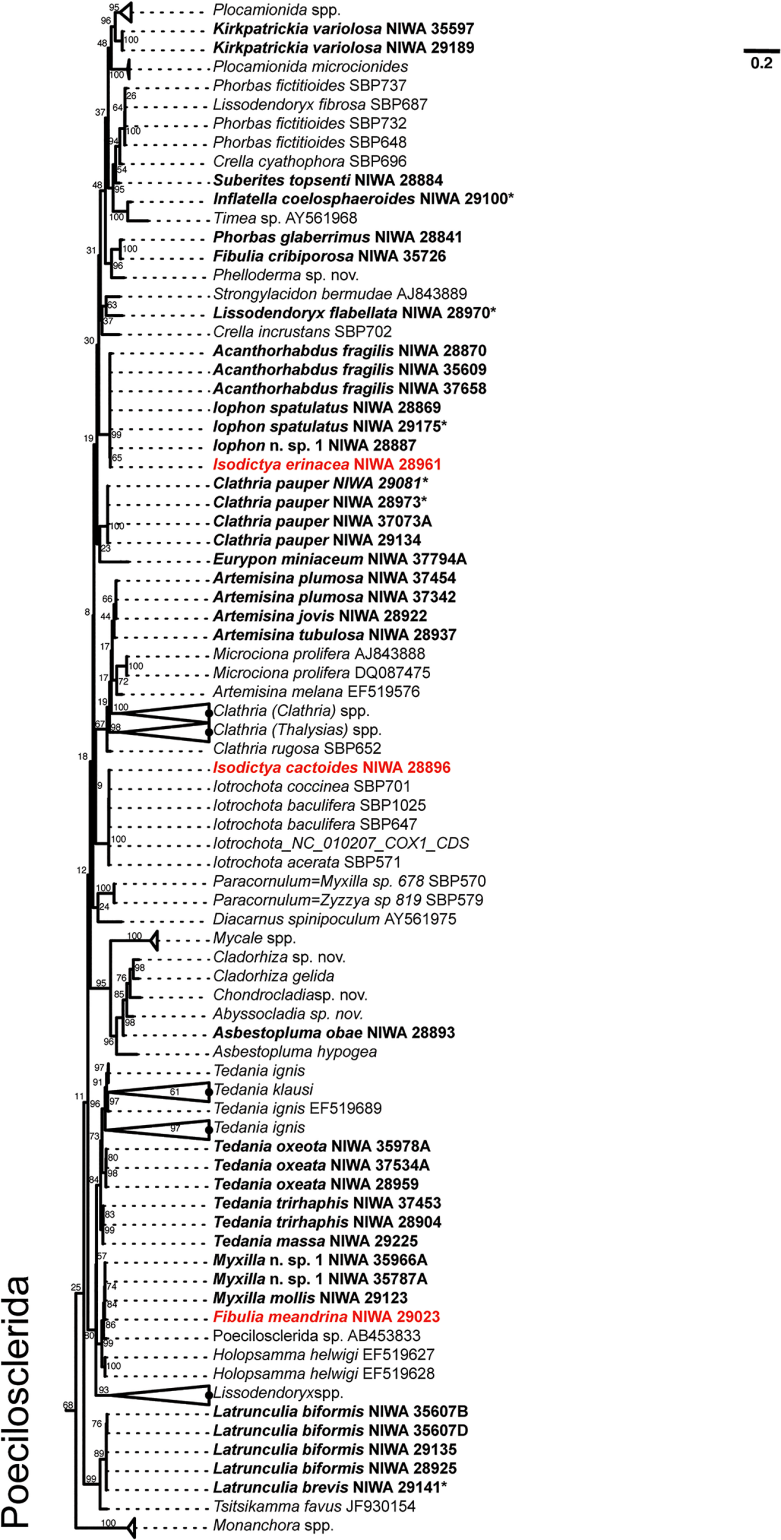
COI maximum likelihood phylogeny of Antarctic sponges (in bold face) belonging Order Hadromerida, Haplosclerida, Halichondrida, Spirophorida, Poecilosclerida (non-chelae bearing). For visualization, subtrees containing Antarctic sponges were pruned from the complete phylogenetic tree that included all sequences analyzed (i.e. GenBank + sequences from this study). Orders are indicated for each subtree. Bootstrap support is given near each branch of the tree. Specimens originally classified as a different species using morphology and reclassified after DNA-barcoding or presenting molecular-morphological discrepancies are in red (see Table 1). Specimens belonging the Spirophorida were sequenced for this study but were already published by Szitenberg et al. 2013.

**Fig 2:**
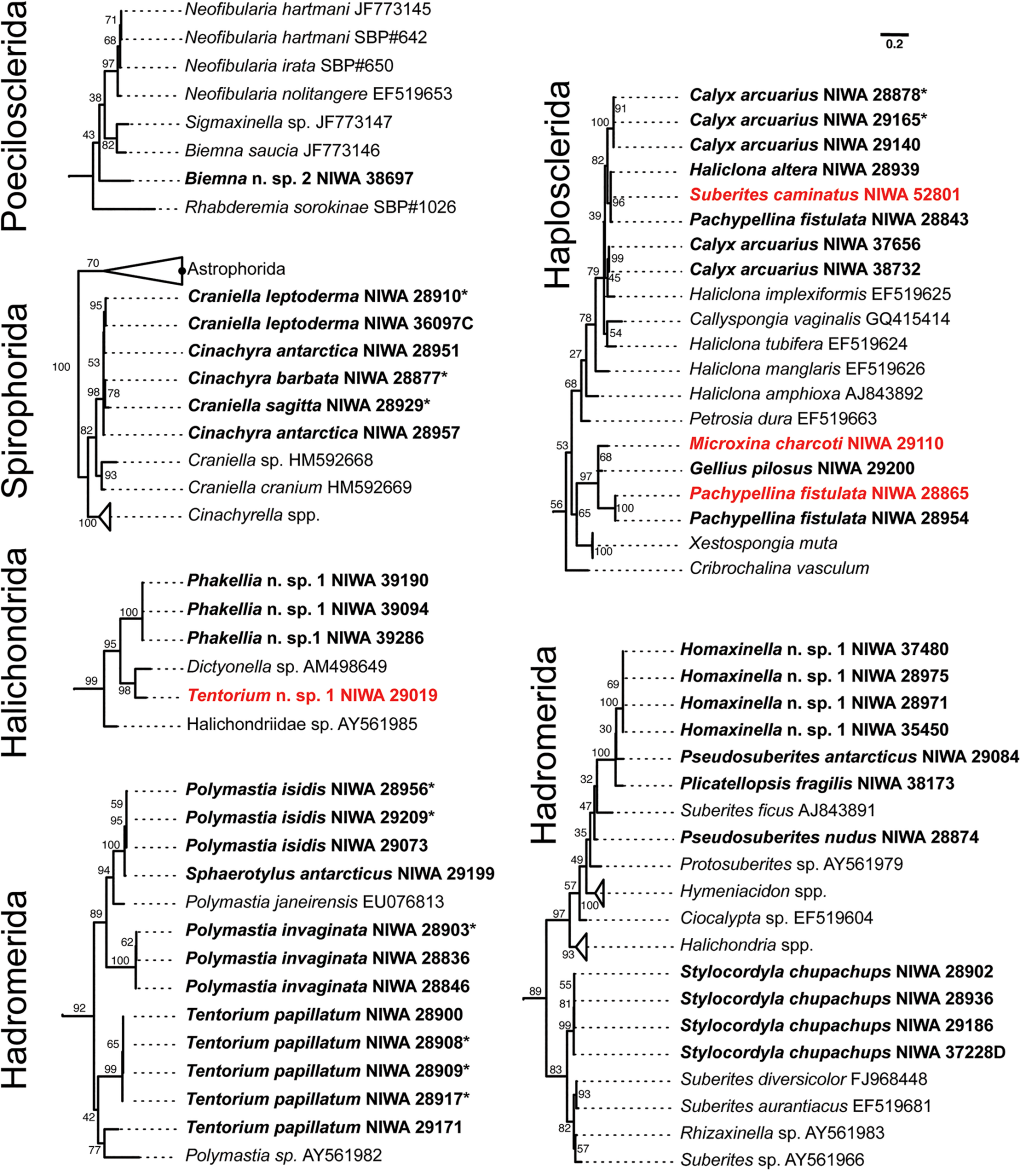
COI maximum likelihood phylogeny of Antarctic sponges (in bold face) belonging Order Poecilosclerida (chelae-bearing). For visualization, subtrees containing Antarctic sponges were pruned from the complete phylogenetic tree that included all sequences analyzed (i.e. GenBank + sequences from this study). Bootstrap support is given near each branch of the tree. Specimens originally classified as a different species using morphology and reclassified after DNA-barcoding or presenting molecular-morphological discrepancies are in red (see Table 1).
